# Evolution of an insect immune barrier through horizontal gene transfer mediated by a parasitic wasp

**DOI:** 10.1371/journal.pgen.1007998

**Published:** 2019-03-05

**Authors:** Ilaria Di Lelio, Anna Illiano, Federica Astarita, Luca Gianfranceschi, David Horner, Paola Varricchio, Angela Amoresano, Pietro Pucci, Francesco Pennacchio, Silvia Caccia

**Affiliations:** 1 Department of Agricultural Sciences, University of Napoli Federico II, Portici (NA), Italy; 2 Department of Chemical Sciences, University of Napoli Federico II, Napoli, Italy; 3 Department of Biosciences, University of Milano, Milano, Italy; Fred Hutchinson Cancer Research Center, UNITED STATES

## Abstract

Genome sequencing data have recently demonstrated that eukaryote evolution has been remarkably influenced by the acquisition of a large number of genes by horizontal gene transfer (HGT) across different kingdoms. However, in depth-studies on the physiological traits conferred by these accidental DNA acquisitions are largely lacking. Here we elucidate the functional role of *Sl gasmin*, a gene of a symbiotic virus of a parasitic wasp that has been transferred to an ancestor of the moth species *Spodoptera littoralis* and domesticated. This gene is highly expressed in circulating immune cells (haemocytes) of larval stages, where its transcription is rapidly boosted by injection of microorganisms into the body cavity. RNAi silencing of *Sl gasmin* generates a phenotype characterized by a precocious suppression of phagocytic activity by haemocytes, which is rescued when these immune cells are incubated in plasma samples of control larvae, containing high levels of the encoded protein. Proteomic analysis demonstrates that the protein *Sl* gasmin is released by haemocytes into the haemolymph, where it opsonizes the invading bacteria to promote their phagocytosis, both *in vitro* and *in vivo*. Our results show that important physiological traits do not necessarily originate from evolution of pre-existing genes, but can be acquired by HGT events, through unique pathways of symbiotic evolution. These findings indicate that insects can paradoxically acquire selective advantages with the help of their natural enemies.

## Author summary

Parasitic wasps are important insect biocontrol agents. These insects are beneficial for the ecological service they provide, which largely contributes to the control of natural populations of their hosts. Paradoxically, they can be beneficial also for non-host individuals attacked by mistake, if these survive after parasitization. In nature, this can happen to lepidopteran larvae when attacked by a wasp harbouring a symbiotic virus that can mediate horizontal gene transfer. Indeed, this virus, injected along with the egg in the body of the host, can get integrated in the genome of the parasitized larva, carrying along exogenous genes. Because the non-host regularly suppresses the parasitoid egg and/or juveniles, any surviving individual with a stable insertion of new genes in the germ line will represent an evolutionary novelty, with expanded functional capacities, if the resulting gene domestication event confers new physiological traits. The immune function here discovered demonstrates that symbiotic associations can drive unique evolutionary pathways, maximizing the fitness of interacting organisms, which evolve as a complex unit with a shared gene pool.

## Introduction

Horizontal gene transfer (HGT) is a mechanism of accidental acquisition of genetic material by means other than reproduction, which in some evolutionary lineages, such as prokaryotes, is considered the major driving force in genome evolution [1]. In theory, all genes may undergo HGT, however current evidence on prokaryotes indicates that housekeeping genes, modulating cellular functions, are significantly more itinerant than regulatory genes [2, 3].

In eukaryotes, HGT occurrence in unicellular organisms has been frequently reported, while, in contrast, it has been considered rare in multicellular organisms, until the advent of high-throughput sequencing technologies, which have allowed the discovery of a considerable number of HGT events in these organisms. Indeed, although less common than in prokaryotes, HGT is far from being of marginal importance in genome evolution of multicellular eukaryotes [4–7)]. The HGT occurrence is frequent both in vertebrates and invertebrates, with bacteria and protists being the major gene donors, as they have established an extensive variety of symbiotic associations with higher organisms, which favours intimate contact and exchange of genetic material [6, 8]. This complements the major role played by transposable elements in shaping genome evolution [5–7, 9].

The study of HGT in eukaryotes has increasingly shed light on its frequency and on the functional categories of the genes involved [3, 5, 7, 9, 10]. However, only a few reports have addressed whether the transferred genes are neutrally included in the genome or functionally integrated into the biological pathways of the recipient organism (i.e., domestication).

Cases of HGT from microorganisms and plants, potentially conferring a functional benefit, have been described in rotifers, nematodes and arthropods [11]. Genome and transcriptome analyses of plant-parasitic nematodes indicate that success in host colonization and exploitation could have been favoured by genes acquired from bacteria through HGT [12–16]. These genes encode plant cell wall-degrading or -modifying enzymes [11–17], as well as enzymes involved in vitamin synthesis [16], or invertases expressed in the nematode digestive system, and likely involved in the metabolism of plant sucrose [13]. Similarly, there is increasing evidence to suggest that HGT plays an active role in modeling insect genomes, providing novel enzymes for digestion and metabolism. Indeed, in coffee berry borer (*Hypothenemus hampei*) [18] and mustard leaf (*Phaedon cochleariae*) [19] beetles, genes acquired from gut bacteria may confer the capacity to hydrolyze host plant polysaccharides; similarly, genes of bacterial and fungal origin may mediate carotenoid biosynthesis in the pea aphid [20] and metabolism of sugar and amino acids in some phytophagous species of Lepidoptera [10]. Moreover, genes conferring the putative capacity to detoxify plant defense chemicals have been acquired by HGT, both in insects and mites [21].

Genes possibly implicated in controlling arthropod immunity or their interactions with other organisms also undergo HGT, as reported for *Drosophila* [5]. Several parasitic chalcidoid wasp species have acquired chitinase genes from fungi, which are expressed in the venom gland to produce virulence factors injected into the host at the oviposition [22]. The recurrent and independent transfer of bacterial genes encoding antibacterial toxins to distinct eukaryotic lineages suggests that these genes can enhance the fitness by adding new immune defense barriers [23]. Only recently it has been demonstrated that these domesticated sequences express active antibacterial effectors in the recipient organisms [24]. Indeed, in the tick *Ixodes scapularis*, this allows the control of the proliferation of the Lyme disease agent, *Borrelia burgdorferi*, in order to keep its abundance at optimal levels for an effective vectoring activity. However, an active role of these genes in the modulation of the immune response against infection is still elusive.

Polydnaviruses (PDV) have recently been identified as a new source of sequences contributing to insect genome evolution by HGT [6, 25]. PDV are viral symbionts of braconid (*Bracovirus*-BV) and ichneumonid (*Ichnovirus*-IV) parasitic wasps attacking lepidopteran larvae, which are injected during the oviposition [26, 27]. They express virulence factors in parasitized hosts triggering immunosuppression and a number of physiological alterations to allow progeny survival and development [26–29]. The PDV are integrated into the wasp’s genome as provirus, while free virions are produced only in the calyx region of the wasp’s ovary. These contain multiple DNA circles, some of which, upon infection of host tissues, become integrated into its genome, starting the expression of virulence factors without undergoing replication [27, 28]. This latter integration property has been proposed as the main route of entrance of genetic material from the BV associated with the parasitic wasp *Cotesia congregata* (*Cc*BV) into the genome of non-permissive hosts, which survive and can transmit *Cc*BV sequences integrated in the germ line [25]. The viral origin of one of these insertions in the moth species *Spodoptera exigua*, named *gasmin*, is unequivocally corroborated by the presence of a bracoviral regulatory sequence [25]. A recent work has tried to analyse the microevolutionary forces driving the domestication of *gasmin* [30]. This was done by baculovirus-mediated expression of gasmin in the larvae of a population lacking a functional *gasmin* gene (i.e., the European population of *S*. *exigua* that bears a truncated non-functional *gasmin* gene). The reduced mortality of larvae infected by a baculovirus expressing *gasmin* was attributed to a putative protecting role exerted by this protein against viral infection and/or replication [30]. However, this presumed antiviral defense barrier was surprisingly associated with an enhanced susceptibility of *S*. *exigua* larvae to bacterial infection [30]. Collectively, the functional evidence provided was largely indirect and strongly influenced by baculovirus infection, which can have effects difficult to tease apart from those induced by gasmin. Then, the microevolutionary scenario cannot be unequivocally interpreted, since a clear conclusion on the effective physiological role of this viral protein and on the adaptive advantage conferred by the domestication of its coding gene is still lacking.

In order to fill this gap, here we report a detailed molecular and functional characterization of a *gasmin* homologue, identified in *S*. *littoralis*, using a gene silencing approach instead of a baculovirus mediated expression in a gasmin-free environment, in order to get rid of all potential experimental artifacts that have apparently influenced previous studies. Our work demonstrates the important role of gasmin in the modulation of the cellular immune response. This sheds new light on the importance that HGT can have in the evolution of metazoan genomes, and on how innate defense barriers in insects can be paradoxically shaped by parasitic wasps and their associated viral symbionts, which are potent pathogens of the wasp’s host.

## Results

### *Spodoptera littoralis* gasmin gene (*Sl gasmin*) and its expression profile

**Fig 1:**
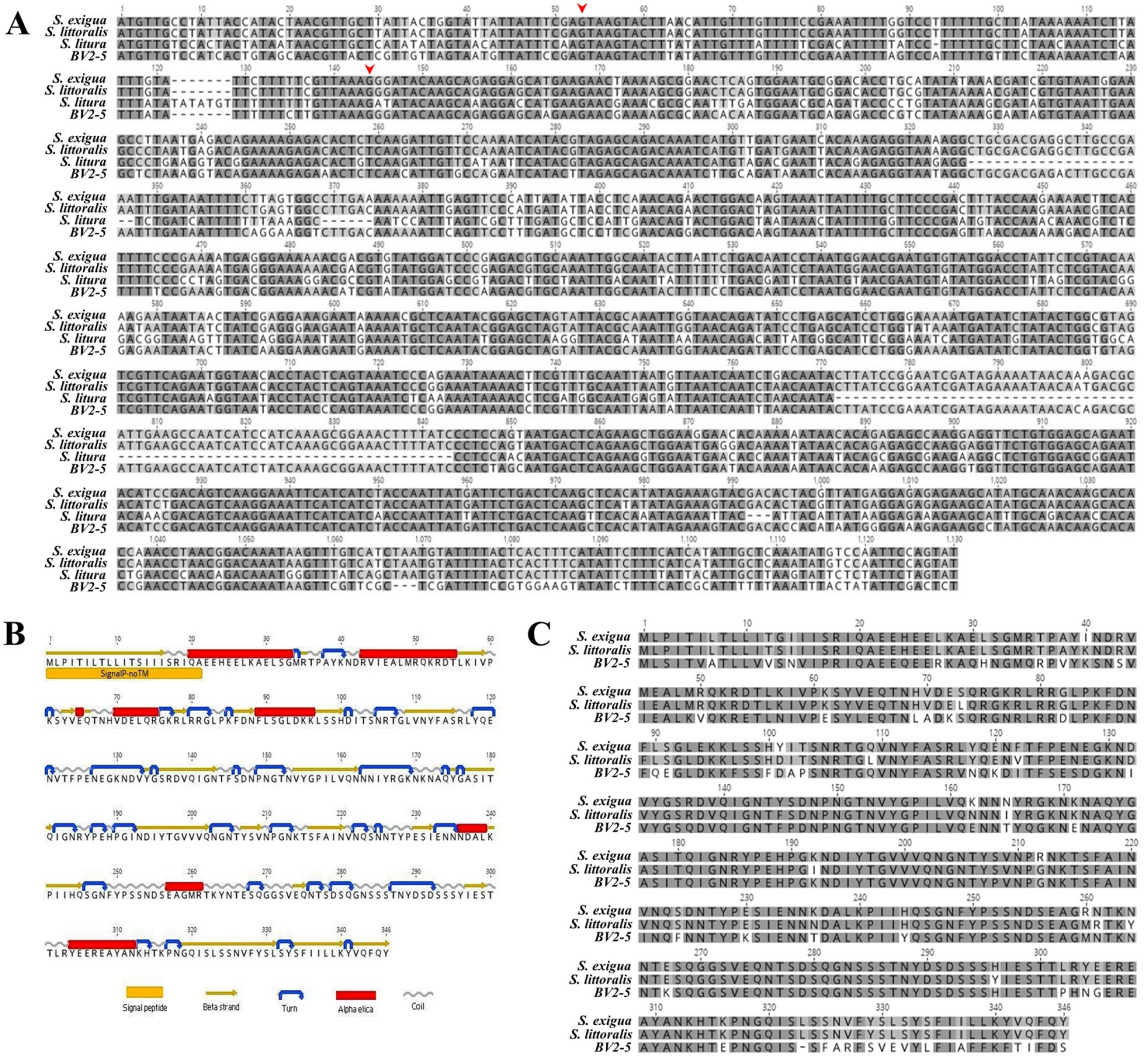
Sequence analysis. DNA sequence alignment of *gasmin* homologues in 3 *Spodoptera* species and in *Cc*BV (*BV2-5*) (the red arrowheads delimit the intronic region) (A), predicted secondary structure (B) and sequence conservation of *Sl* gasmin (C). Secondary structure prediction of *Sl* gasmin was carried out with the EMBOSS: Garnier algorithm; the Inter ProScan tool identified a potential signal-peptide. Bioinformatics analyses were performed using Geneious v6.1.6 (Biomatters, available from www.geneious.com) (A and C). DNA and protein alignments were performed using the Clustal W algorithm; black and grey shadings indicate identity and high conservation of amino acids, respectively.

The genomic sequence of *Sl gasmin*, including the intron (1126 bp), shows very high identity with *S*. *exigua gasmin* (KP406767), *S*. *litura gasmin* (obtained through data mining; MTZO01009970.1) and with viral *BV2-5* (AJ632326) (98, 72 and 86% respectively) (Fig 1A). The coding DNA sequence (CDS) encodes a predicted protein of 346 amino acids (aa) (with a putative signal peptide of 21 aa) (Fig 1B and 1C), that shows 95% and 76% sequence identity with *S*. *exigua* gasmin and with the homologue viral protein encoded by *Cc*BV (*Cc*BV 25.3; CAG17487), respectively (Fig 1B and 1C).

**Fig 2:**
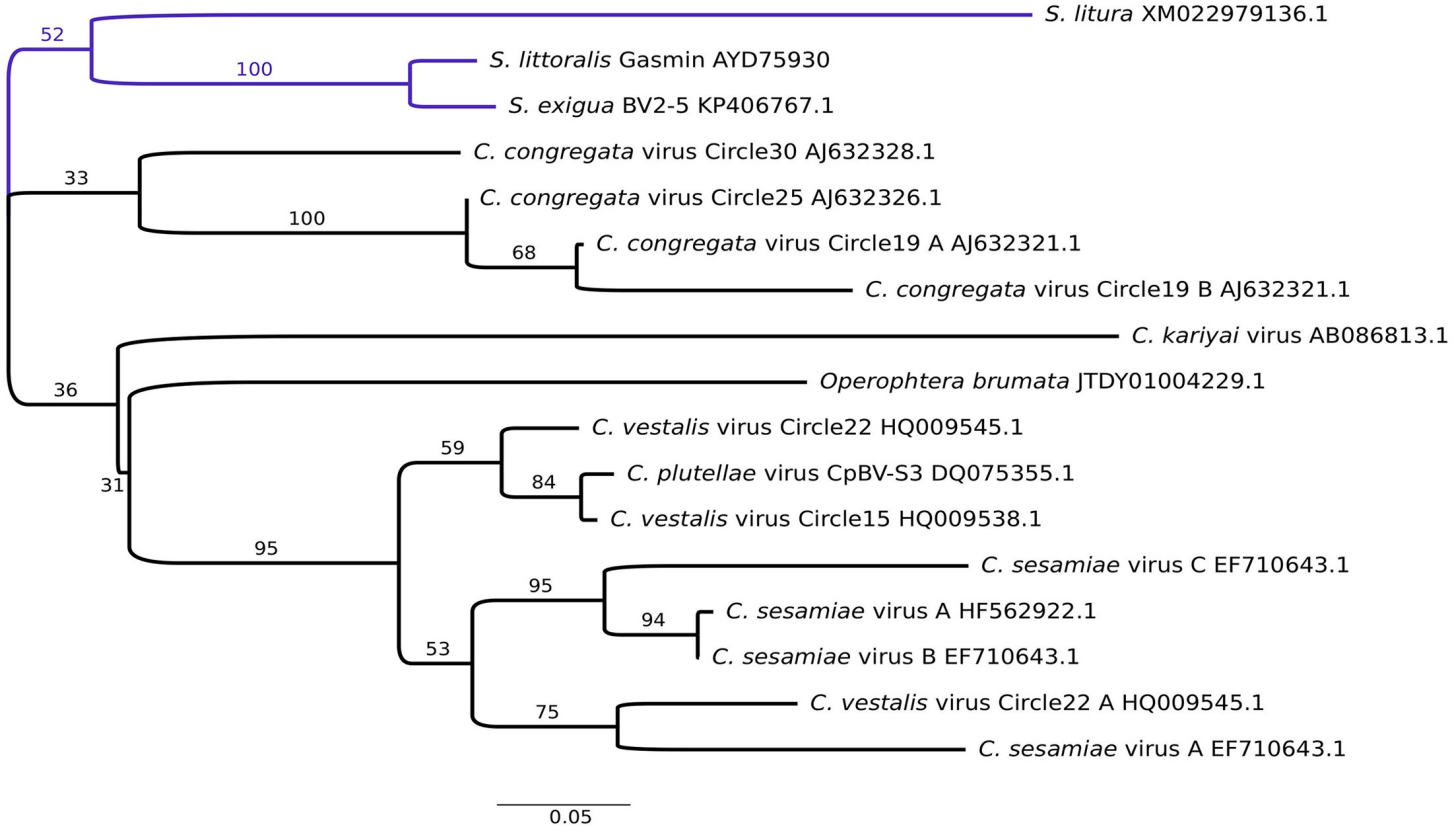
Molecular phylogenetic tree of gasmin-like proteins. The evolutionary history was inferred for aligned sequences by the Maximum Likelihood method, under the JTT amino acid substitution matrix. The sequence identity of *Spodoptera littoralis* gasmin with the retrieved protein sequences was higher than 60% in all cases, except for *S*. *litura* (57%) and *Cotesia kariyai* bracovirus (47%). Query cover was above 94%, except for *C*. *vestalis* virus circle 22 (79%) and *C*. *kariyai* bracovirus (63%). The numbers above the branches denote the bootstrap proportions.

To learn more about the origin of the *Sl gasmin*, we retrieved all sequences showing high similarity, available in NCBI, and constructed a similarity tree using the predicted amino acid sequences (Fig 2). Phylogenetic analyses strongly support the monophyly of the *S*. *exigua* and *S*. *littoralis* protein sequences (BS = 100), and indicate that *S*. *litura* represents a sister to these sequences. Other relationships, including the placement of a homologue identified in the winter moth (*Operophtera brumata*), are poorly supported and available sampling does not permit the confident assignment of likely donors for any of the lepidopteran genes. While all of the lepidopteran proteins (with the exception of the *S*. *exigua* and *S*. *littoralis* sequences) are highly divergent from one-another, examination of the aligned protein sequences reveals that most indels are concentrated in regions that also show indels and high divergence among viral sequences, possibly consistent either with rapid evolution under altered selective constraints in various lepidopteran groups, or with multiple acquisitions and independent losses in insects. Further sequence sampling would be required to conclusively resolve this issue.

To investigate the existence of selective pressure on *S*. *littoralis* and *S*. *exigua* sequences we used FEL (Fixed Effects Likelihood), a program which uses a maximum-likelihood approach to infer nonsynoymous (dN) and synonymous (dS) substitution rates on a per-site basis for a given coding alignment and corresponding phylogeny. As expected, negative purifying selection has been observed at 26 sites with *P* values≤0.05 (S1 File).

**Fig 3:**
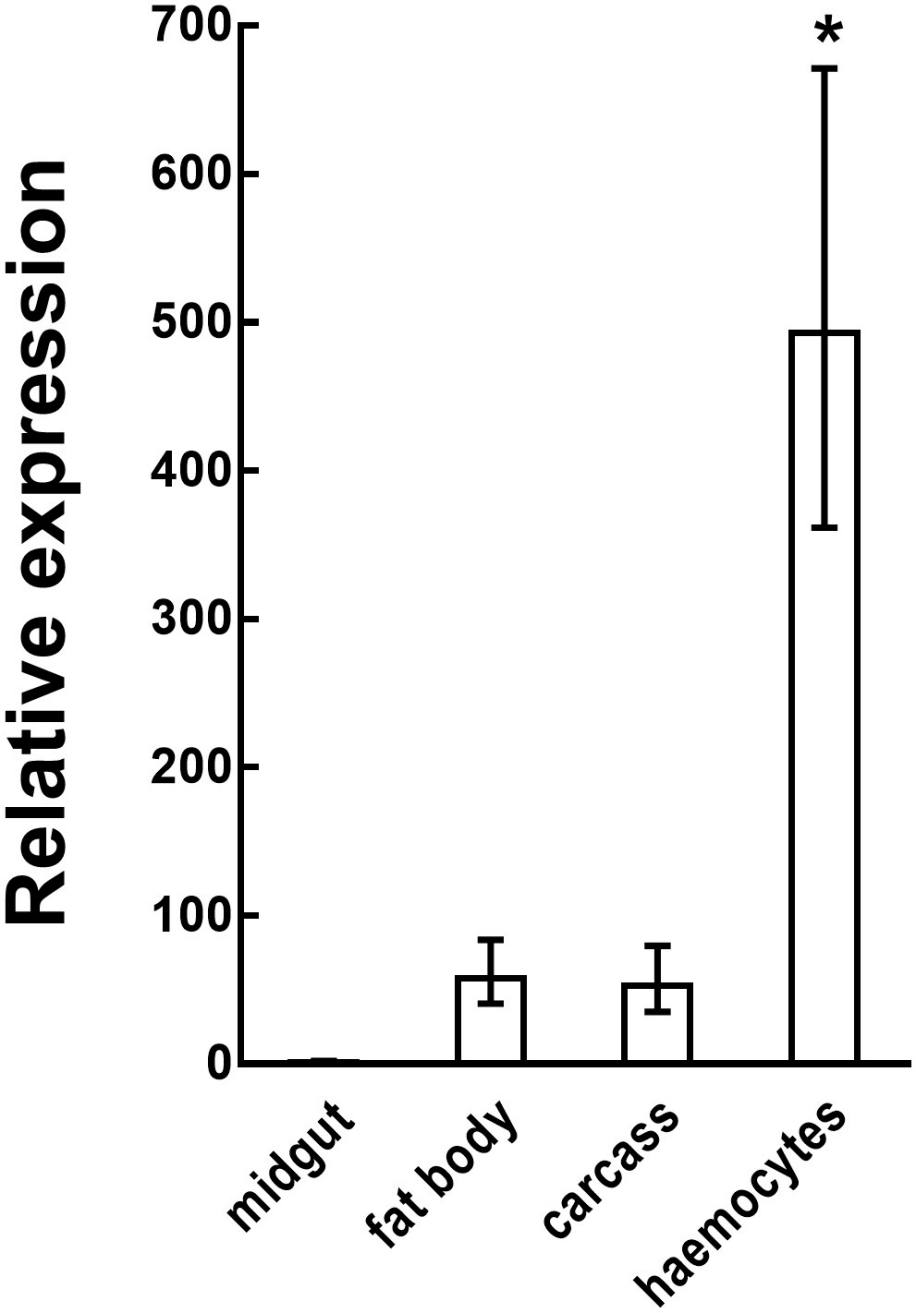
*Sl gasmin* transcript level in different tissues of *Spodoptera littoralis* larvae. *Sl gasmin* relative expression, determined by qRT-PCR, was significantly higher in the haemocytes compared with the other tissues analyzed. *S*. *littoralis β-actin* was used as reporter gene. The values reported are the mean ± S.E. (*P<0.001, One-Way ANOVA followed by Bonferroni’s test, n = 7).

The expression profile of *Sl gasmin* in different tissues of *S*. *littoralis* larvae was analyzed by qRT-PCR. The haemocytes (i.e., the circulating immune cells) were by far the most active site of transcription (One-Way ANOVA: F_(3,24)_ = 49.07, n = 7, *P*<0.0001, df = 27) (Fig 3), suggesting a key-role of this gene in the immune response.

**Fig 4:**
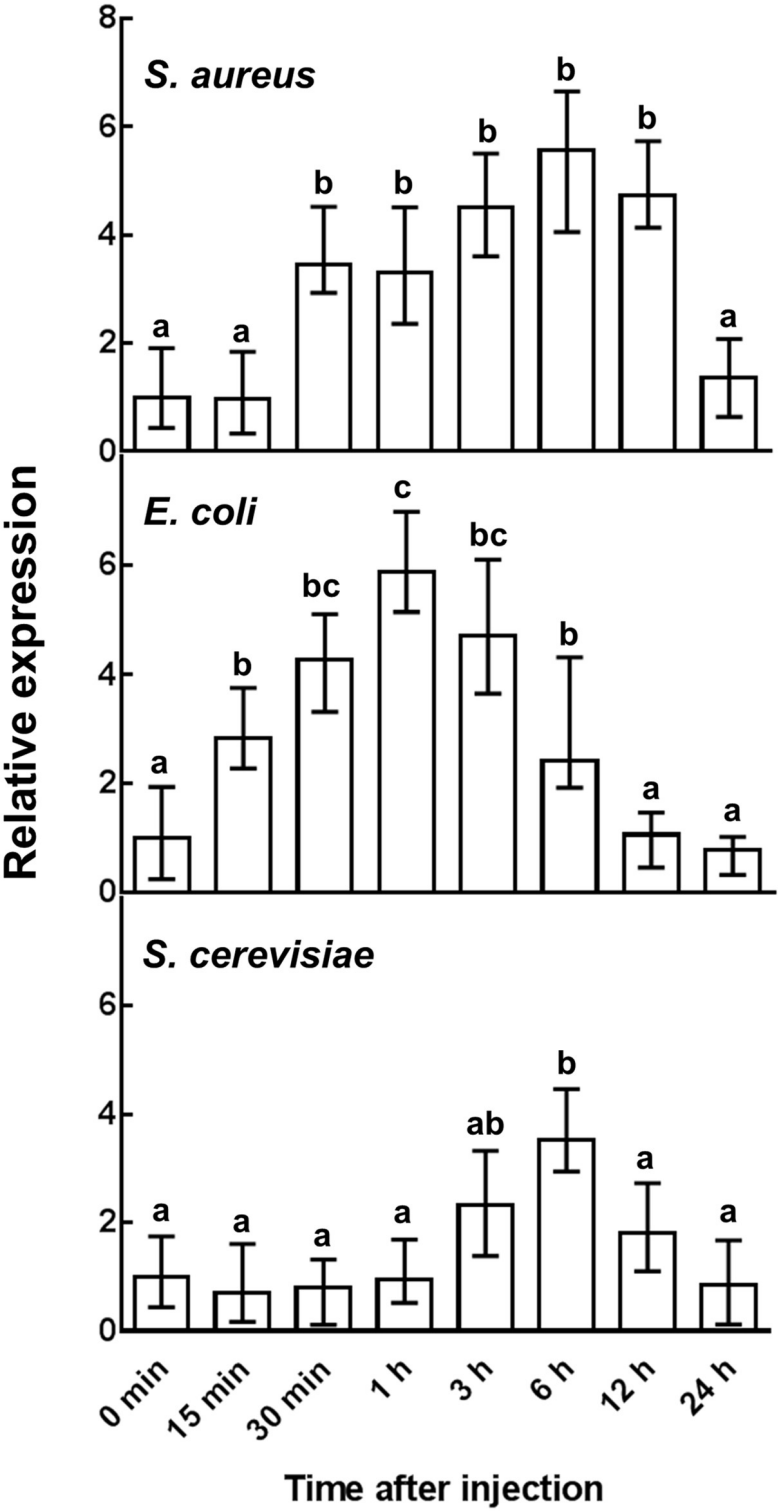
Relative expression level of *Sl gasmin* after microbial challenge. The transcription level of *Sl gasmin*, determined by qRT-PCR, was significantly up-regulated in *Spodoptera littoralis* larvae by injection of the Gram-positive bacterium *Staphylococcus aureus*, the Gram-negative bacterium *Escherichia coli*, and the yeast *Saccharomyces cerevisiae*. *S*. *littoralis β-actin* was used as reporter gene. Data points are the mean ± S.E. of 3 biological replicates. Different letters above each bar indicate significant differences (*P*<0.05, One-Way ANOVA followed by Bonferroni’s test).

Because this gene is of viral origin, and its viral homologue is expressed in parasitized hosts, we formulated the hypothesis that it confers a selective advantage both to the wasp bearing the donor viral symbiont and to the recipient moth species. Indeed, because the protection of wasp’s juveniles against host immune responses is mediated by broad immunosuppressive strategies, it is reasonable to assume that a reinforcement of the antimicrobial barriers, by preventing secondary infections of the host, is also beneficial for the wasp progeny. To corroborate this hypothesis, *Sl gasmin* transcription was assessed in response to microbial challenge. The injection of both Gram-positive (*Staphylococcus aureus*) and Gram-negative (*Escherichia coli*) bacteria in *S*. *littoralis* larvae, as well as of the yeast *Saccharomyces cerevisiae*, significantly enhanced the transcription of *Sl gasmin* in the haemocytes (One-Way ANOVA *S*. *aureus*: F_(7,88)_ = 150.68, *P*<0.0001, n = 12, df = 95; *E*. *coli*: F_(7,88)_ = 169.61, *P*<0.0001, n = 12, df = 95; *S*. *cervisiae*: F_(7,88)_ = 68.072, *P*<0.0001, n = 12, df = 95), with slightly different temporal profiles (Fig 4). The expression level of the target gene increased very rapidly following bacteria injection, while *S*. *cerevisiae* challenge triggered a comparatively slower response soon after injection, followed by a gradual decrease to the basal level 12 h post-injection (Fig 4).

### Functional analysis of *Sl gasmin* by RNAi

**Fig 5:**
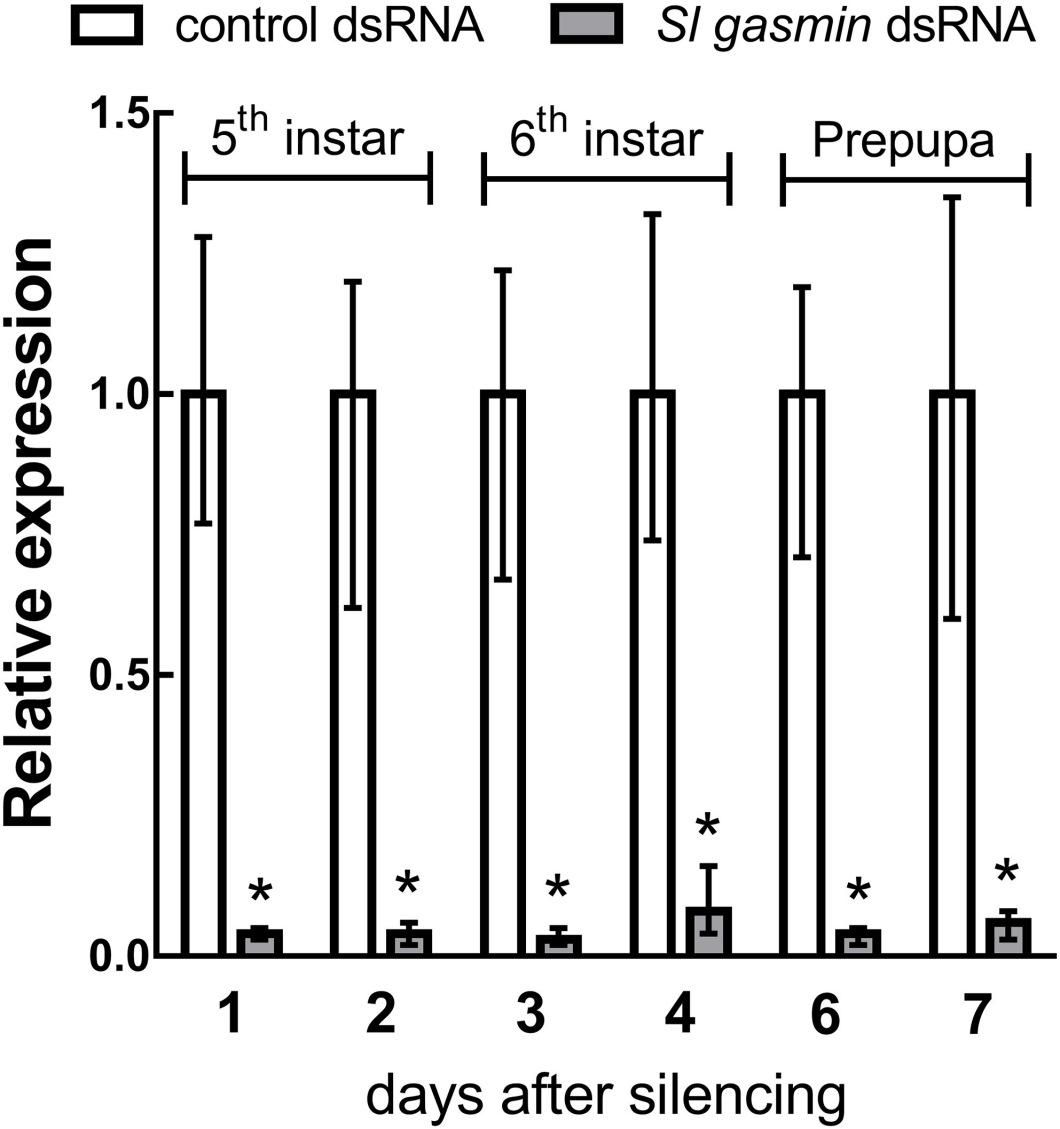
*Sl gasmin* silencing in *Spodoptera littoralis* larvae. After oral administration of control or *Sl* g*amin* dsRNA during 4^th^ instar, the expression of *Sl gamin*, determined by qRT-PCR was significantly down-regulated until pupation. *S*. *littoralis β-actin* was used as reporter gene. The values reported are the mean ± S.E. (*P<0.0001, Student’s *t* test).

The very high transcription level in the haemocytes and its enhancement following microbial challenge strongly suggested the important role for *Sl gasmin* in the immune response mounted by *S*. *littoralis* larvae. To characterize *Sl gasmin* at the functional level, we pursued a loss-of-function strategy, through RNAi-mediated silencing, which proved to be very efficient in *S*. *littoralis* [31, 32], like in many other lepidopteran species [33–39], in spite of several cases of refractiveness to RNAi reported for species/strains belonging to this order [39]. Oral treatment of experimental larvae with *Sl gasmin* dsRNA was effective in silencing the target gene from the first day of the 5^th^ instar until the prepupal stage (Student’s *t* test. Day 1: *t* = 26.838, df = 14, *P*<0.0001; day 2: *t* = 11.362, df = 14, *P*<0.0001; day 3: *t* = 15.408, df = 14, *P*<0.0001; day 4: *t* = 18.456, df = 14, *P*<0.0001; day 6: *t* = 22.903, df = 14, *P*<0.0001; day 7: *t* = 15.258, df = 14, *P*<0.0001) (Fig 5).

**Fig 6:**
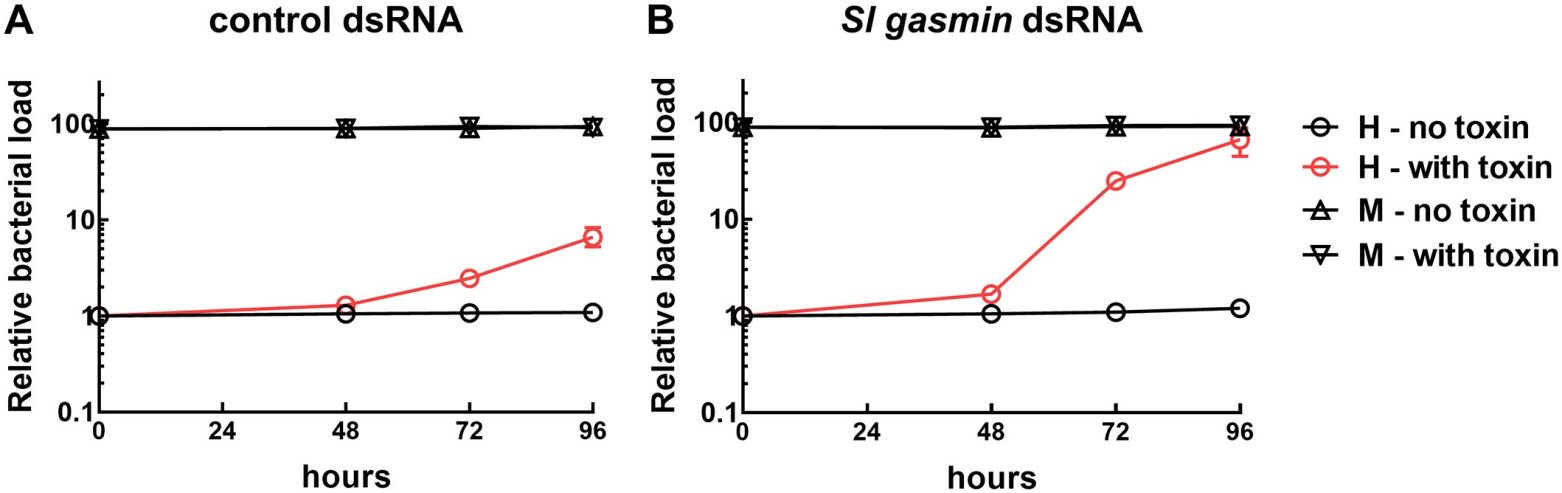
Relative quantification of bacterial load by qRT-PCR. Relative change over time of the bacterial load in *Spodoptera littoralis* larvae, exposed to control dsRNA (A) or *Sl gasmin* dsRNA (B) and fed with artificial diet on which a solution of *Bacillus thuringiensis* Cry1Ca toxin (2.7 μg/cm^2^) was layered. The bacterial load in the haemolymph (H, red lines with empty circles) resulted significantly influenced by toxin treatment, both in control and silenced larvae, with these latter showing a much higher bacterial load increase over time (see S1 Table for statistics), whereas no significant changes were observed in the midgut (M).

The relevance of the immune role played by *Sl* gasmin *in vivo* was assessed by scoring the impact of the silencing of its coding gene on the host septicaemia induced by *Bacillus thuringiensis* toxin Cry1Ca, using the experimental approach previously described to study the *Bt* killing mechanism [32]. Cry toxins produced by this entomopathogenic bacterium are active upon ingestion. When in the midgut, they interact with brush border membranes of epithelial cells and cause the osmotic lysis of these latter. The resulting tissue lesions allow the entrance of gut microflora into the insect haemocoel, causing septicaemia and insect death [32]. Then, we hypothesized that the reduced immunocompetence associated with *Sl gasmin* silencing should result in the enhancement of Cry1Ca killing activity, as a consequence of uncontrolled bacterial proliferation. Indeed, this was the case. *Sl gasmin* dsRNA treatment significantly enhanced the mortality of *S*. *littoralis* larvae treated with Cry1Ca toxin (Table 1), and this enhancement was associated with a significant increase of bacterial load in the haemolymph (Fig 6, see S1 Table for statistics). These data clearly support the key-role played by this gene in the modulation of the antimicrobial immune response *in vivo*, and nicely corroborate the proposed key-role of septicaemia in the *Bt* killing mechanism [32].

**Table 1:** Enhancement of Cry1Ca toxicity by host immunosuppression.

Treatment^a^	LC_50_^b^		TI^c^
	Control dsRNA	*Sl gasmin* dsRNA	
Cry1Ca	6.8 (4.5–10.0)	2.7 (1.6–4.3)	2.5 (1.2–5.2)

^a^Toxicity of Cry1Ca toxin was assessed on *Spodoptera littoralis* larvae after RNAi-mediated silencing of the immune gene *Sl gasmin* (*Sl gasmin* dsRNA), and in immune competent controls (control dsRNA).

^b^Concentration (μg Cry1Ca/cm^2^ diet) killing 50% of experimental larvae, with 90% fiducial limits reported in parentheses.

^c^The toxicity increase (TI) is calculated as the ratio between LC_50_ values scored in *control* dsRNA control larvae, and in larvae treated with *Sl gasmin* dsRNA; 95% fiducial limits are reported in parentheses.

**Fig 7:**
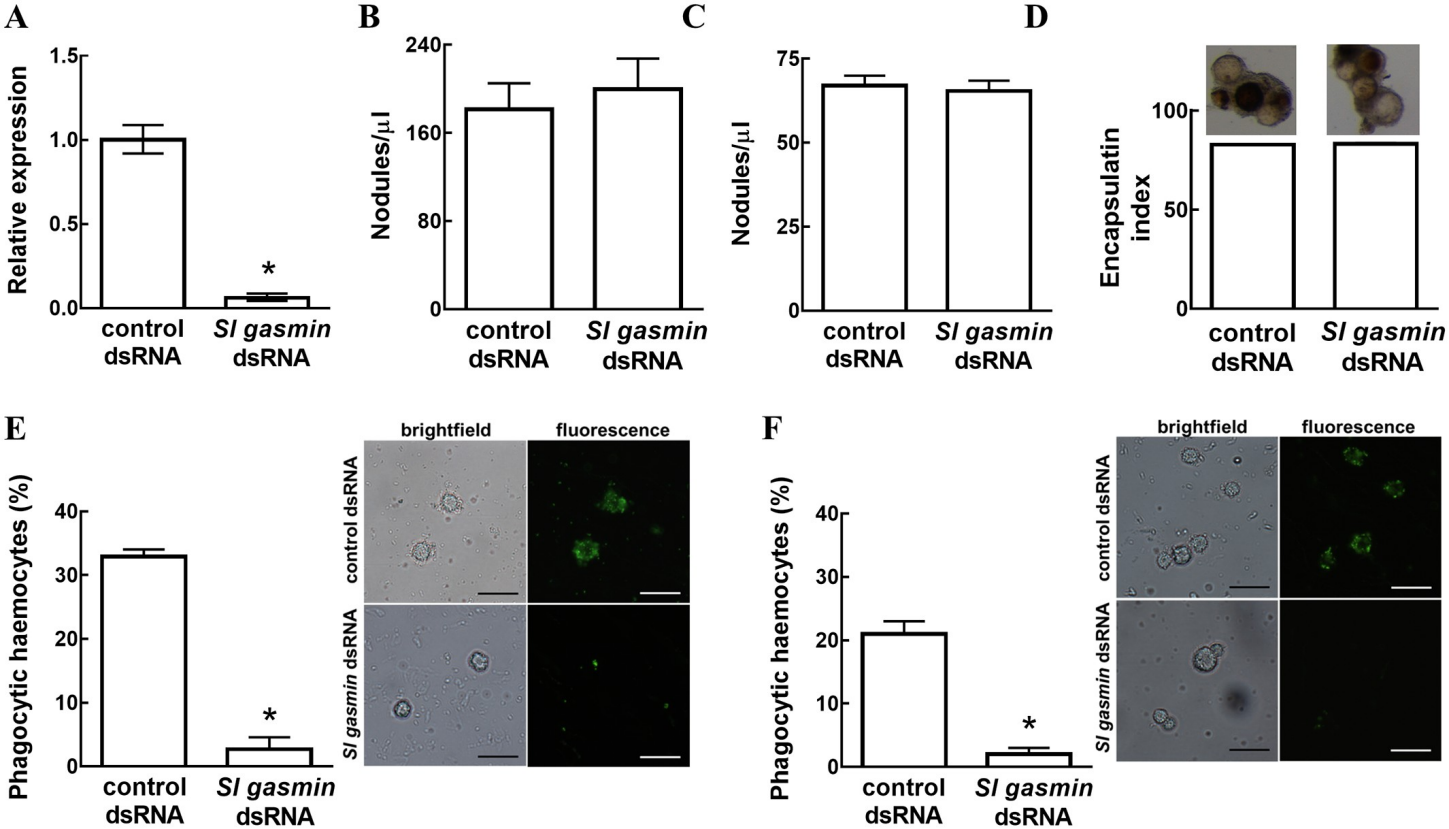
Cellular immune responses by *Spodoptera littoralis* larvae as affected by RNAi mediated silencing of the gene *Sl gasmin*. The oral administration of *Sl gasmin* dsRNA induced a significant transcriptional down-regulation of the target gene in the haemocytes of *S*. *littoralis* (A). *S*. *littoralis β-actin* was used as reporter gene. Gene silencing did not influence nodulation of the Gram-negative bacterium *Escherichia coli* (B) and of the yeast *Saccharomyces cerevisiae* (C). Chromatography beads injected into *S*. *littoralis* larvae orally treated with *Sl gasmin* dsRNA were regularly encapsulated and melanized as in controls (D). Conversely, RNAi mediated silencing of *Sl gasmin* significantly reduced the phagocytic capacity of haemocytes against Gram-negative (*E*. *coli*) (E) and Gram-positive (*Staphyloccus aureus*) (F) bacteria. The values reported are the mean ± S.E. (*P<0.001, Student’s *t* test). Bars: 15 μm.

Thereafter, we performed specific experiments to understand which component of the immune response was controlled by *Sl gasmin*. We focused on the three main cellular responses against foreign invaders that are mediated by haemocytes: nodulation, encapsulation and phagocytosis [40–41]. On the occasion of each experiment performed to assess the impact of *Sl* gasmin silencing on immune response, we always checked the level of gene silencing on day 1 of 5^th^ instar larvae (*t* = 37.873, *P*<0.0001, df = 46, n = 24) (Fig 7A).

Gene silencing did not interfere with the nodulation response (i.e., multicellular aggregation of haemocytes that entrap a large number of microorganisms) induced against either bacteria (Fig 7B) or yeast (Fig 7C) (*E*. *coli*: *t* = 0.501, *P* = 0.6208, df = 26, n = 14; *S*. *cervisiae*: *t* = 0.386, *P* = 0.7028, df = 26, n = 14). Similarly, encapsulation (i.e., formation of a multilayered capsule of haemocytes around the non-self object) and melanization of chromatographic beads injected into experimental larvae were not affected by gene silencing (*t* = 0.674, *P* = 0.5056, df = 22 n = 14) (Fig 7D).

In contrast, phagocytosis of bacteria was strongly inhibited in experimental larvae treated with *Sl gasmin* dsRNA, as their haemocytes were almost completely unable to internalize either Gram-negative (*E*. *coli*) (*t* = 13.610, *P*<0.0001, df = 19, n = 11) (Fig 7E) or Gram-positive (*S*. *aureus*) bacteria (*t* = 10.725, *P*<0.0001, df = 20, n = 11), within 10 min or 30 min of incubation, respectively (Fig 7F). We focused on such a short time in order to detect any precocious enhancement of one of the immune barriers most rapidly activated by foreign invaders [41–43].

**Fig 8:**
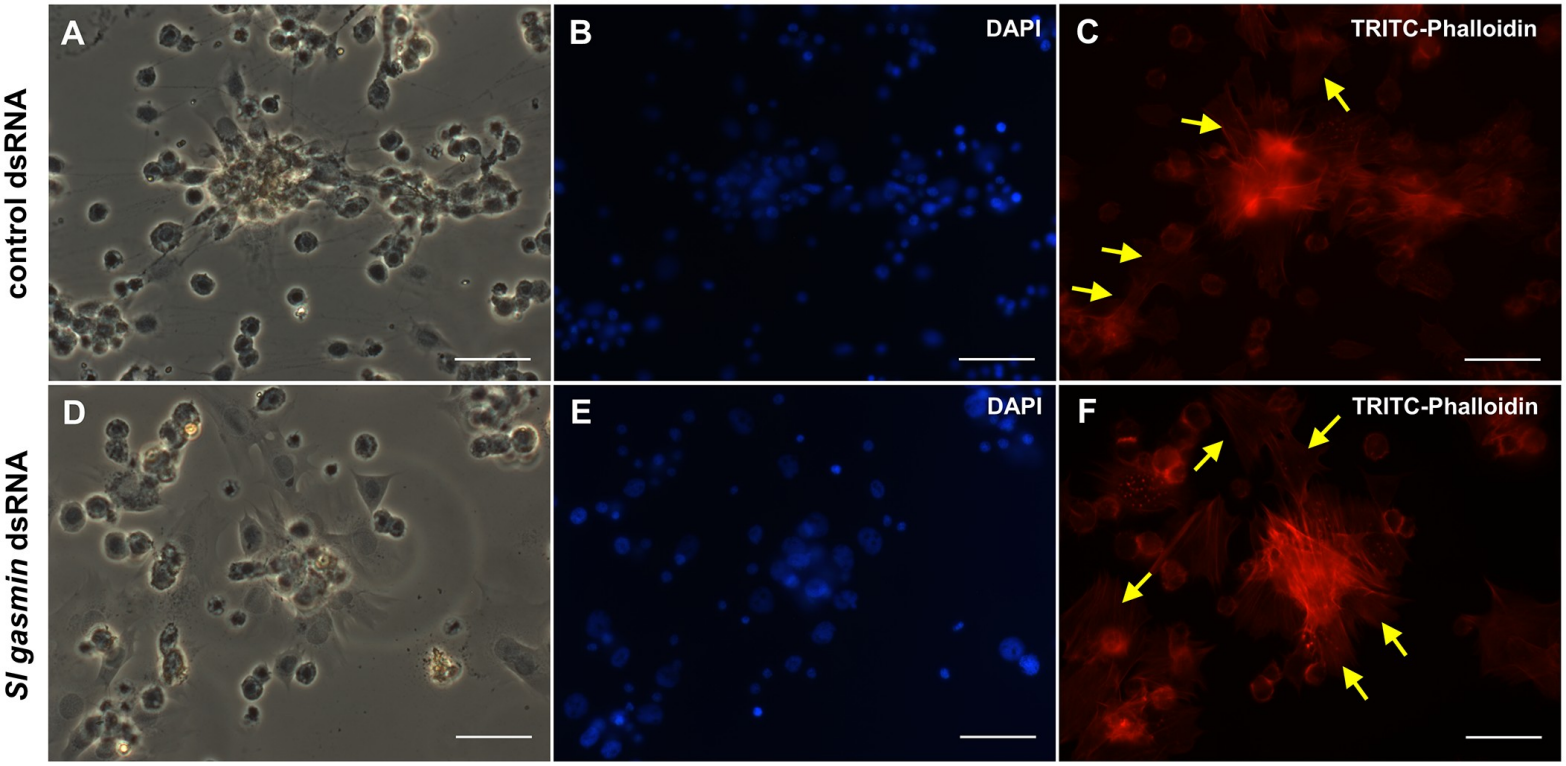
*In vitro* behaviour and actin cytoskeleton of *Spodoptera littoralis* haemocytes. Bright-field images (A, D), DAPI (nuclear DNA) (B, E) and TRITC-Phalloidin (F-actin) staining (C, F) of haemocytes extracted from *S*. *littoralis* larvae, orally treated with control (*GFP* dsRNA) or *Sl gasmin* dsRNA. Different types of haemocytes from experimental and control larvae adhered and spread on glass slides singly or in clusters (A, D), showing similar actin networks of polymerized actin; lamellipodia are denoted with yellow arrows (C, F). Bars: 30 μm.

To check if the disruption of phagocytosis was due to a negative effect of gene silencing on cytoskeleton architecture and dynamics, the haemocyte spreading/adhesion and the distribution/polymerization of actin were investigated, as readouts of their immunocompetence. Haemocytes extracted from both control and silenced larvae showed identical levels of aggregation and adhesion on glass surfaces (Fig 8A and 8D), large nuclei (Fig 8B and 8E), and a similar network of polymerized actin, underlying plasma membrane protrusions, such as lamellipodia (sheet-like protruding two-dimensional lobes of the plasma membrane at the leading edge of the cell) and filopodia (protruding one-dimensional microspikes of the cell membrane) extensions (Fig 8C and 8F). These findings demonstrated that the adhesion properties of haemocytes, which strongly depend upon proper actin cytoskeletal dynamics, were completely unaffected by gene silencing.

**Fig 9:**
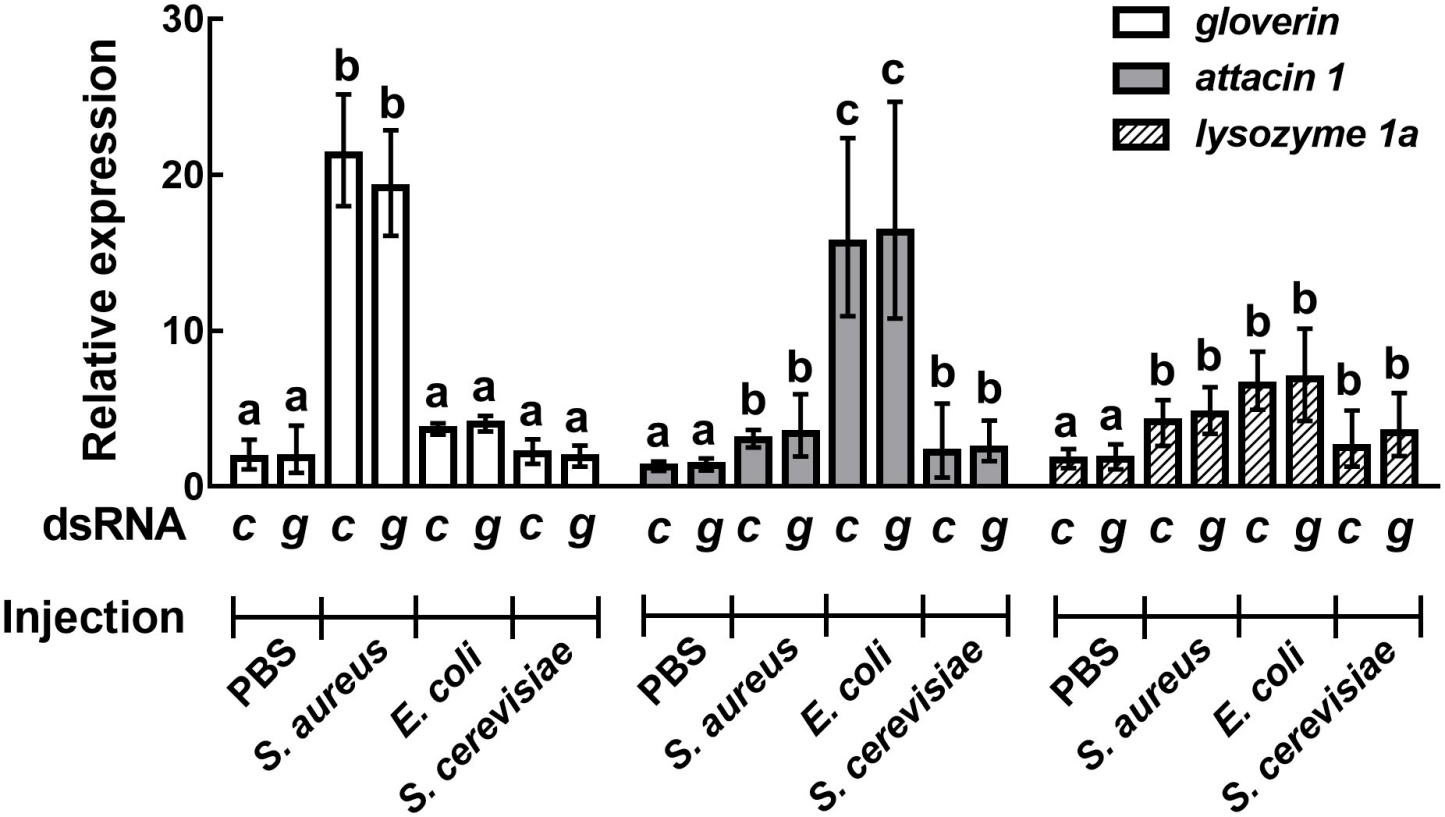
Humoral immune response by *Spodoptera littoralis* larvae as affected by RNAi mediated silencing of the immune gene *Sl gasmin*. Immune challenge with Gram-positive (*Staphyloccus aureus*), Gram-negative (*Escherichia coli*) bacteria or with the yeast *Saccharomyces cerevisiae* was performed on *S*. *littoralis* larvae after oral administration of control (c) or *Sl gasmin* (g) dsRNA. After 3 hours from the immune challenge, the transcript level of genes encoding the humoral effectors was significantly enhanced (*attacin 1*: F_(3,126)_ = 68.13, *P*<0.0001; *gloverin* F_(3,126)_ = 184.16, *P*<0.0001; *lysozyme 1a* F_(3,126)_ = 33.53, *P*<0.0001), but was not influenced by gene silencing (see S1 Table for statistics). *S*. *littoralis β-actin* was used as reporter gene. The values reported are the mean ± S.E. Different letters denote significant differences between treatments, within each gene considered.

To assess whether the *Sl gasmin* silencing had any impact on *S*. *littoralis* humoral immune responses, we analyzed the transcription profiles of genes encoding humoral effectors secreted by haemocytes in the haemolymph, including antimicrobial peptides (AMP) and lysozyme [44, 45], following immune challenge with different microorganisms. No negative effects of *Sl gasmin* silencing on the ability of haemocytes to mount a humoral response were detected. Indeed an increase of transcript level of humoral effectors upon immune challenge was observed both in silenced and control larvae (Fig 9, see S1 Table for statistics).

Collectively, these results indicate that *Sl gasmin* exerts a key-role in the modulation of phagocytosis by larval haemocytes.

### *Sl* gasmin is an opsonizing factor

The presence of a signal peptide in *Sl* gasmin suggested that it is likely secreted by haemocytes and exerts its role in the haemolymph plasma. To investigate this hypothesis, the putative presence of *Sl* gasmin in the haemolymph plasma and its changes in response to gene silencing were assessed by liquid chromatography-tandem mass spectrometry in the multiple reaction mode (LC-MRM/MS). Specific peptides belonging to *Sl* gasmin sequence were selected (S2 Table) and monitored by MRM analysis of the tryptic digest of haemolymph samples, leading to the unambiguous identification of the protein.

**Fig 10:**
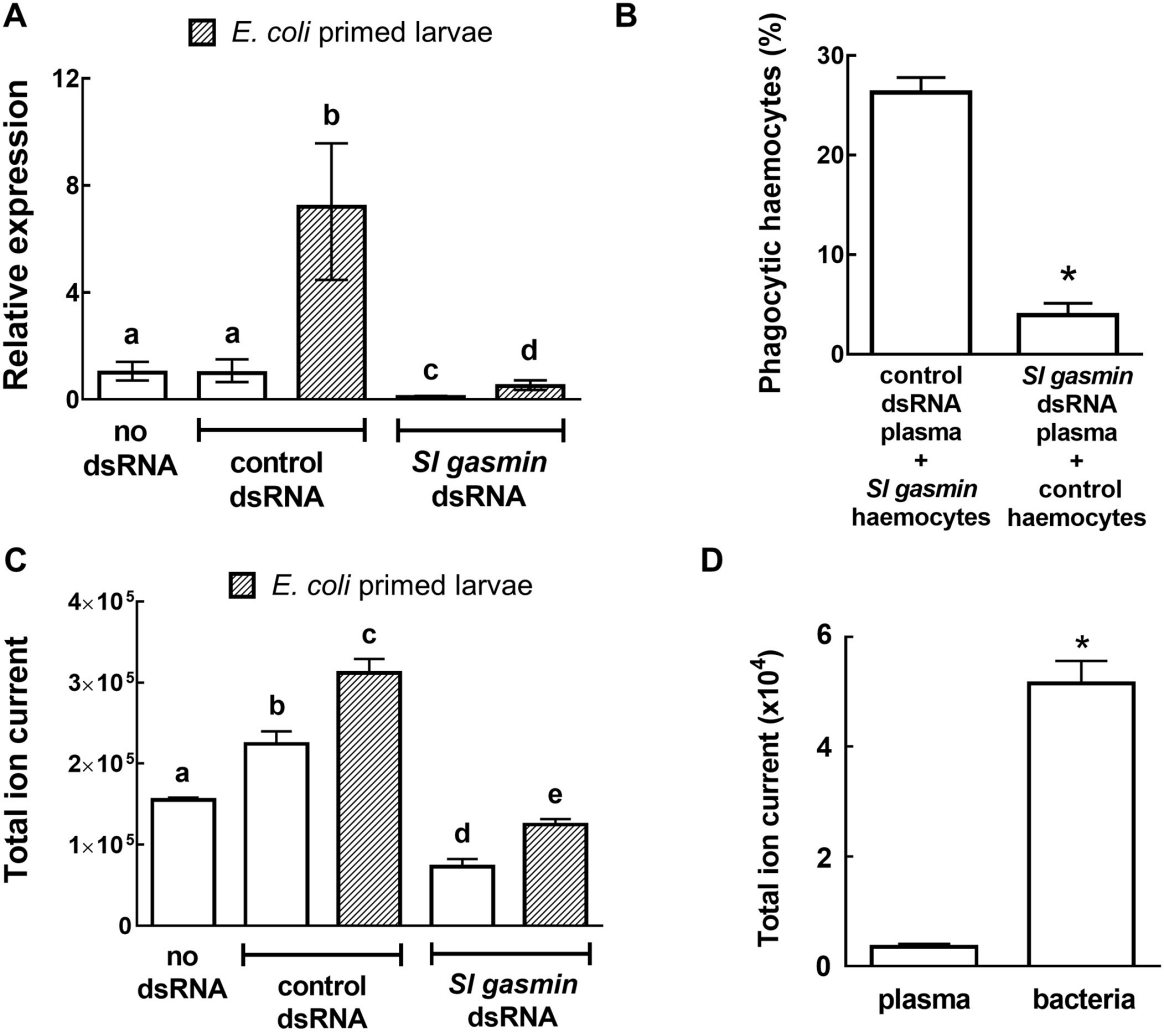
Phagocytosis by *Spodoptera littoralis* larvae as affected by the presence in the plasma of the opsonizing factor *Sl* gasmin. RNAi mediated silencing of the gene *Sl gasmin* significantly reduced its transcription level, determined by qRT-PCR (A) and the presence of the encoded protein in the haemolymph plasma, determined by LC-MRM/MS (C), both under basal and immune challenged conditions. The presence of *Sl* gasmin in the incubation medium was essential to promote phagocytosis *in vitro* of fluorescent bacteria (*Escherichia coli*), as supported by the functional rescue of haemocytes unable to perform phagocytosis, obtained from *Sl gasmin* silenced larvae, when transferred to plasma of control larvae (B). The opsonizing role of *Sl* gasmin was demonstrated by LC-MRM/MS measurements of its amount recovered by proteolytic shaving of *E*. *coli* cells incubated in presence of haemolymph plasma (D). *S*. *littoralis β-actin* was used as reporter gene. The values reported are the mean ± SE. Different letters above each bar, in A and C, indicate significant differences (*P*<0.05, based on an One-Way ANOVA followed by Bonferroni’s test; *P<0.001, Student’s *t* test, in B and D).

The observed changes of *Sl gasmin* transcript level induced by gene silencing (One-Way ANOVA: F_(4,40)_ = 401.6, *P*<0.0001, n = 9, df = 44) (Fig 10A) were perfectly mirrored by changes in the abundance of the protein in the haemolymph plasma (Kruskal-Wallis test: KW = 36.28, *P*<0.0001, n = 8) (Fig 10B). Moreover, the injection of *E*. *coli* into control larvae induced a steep increase of *Sl gasmin* transcription (Fig 10A) and of the encoded protein titer in the haemolymph plasma (Fig 10B).

The presence of *Sl* gasmin protein in the haemolymph plasma, associated with (1) the increase of its titer triggered by immune challenge, and (2) the phagocytosis failure in its absence strongly suggested a functional role of *Sl* gasmin in mediating pathogen recognition and subsequent phagocytosis by haemocytes. Indeed, *Sl* gasmin could act as an opsonization factor, i.e., a molecule that coats pathogens and mediates their recognition and suppression by immune cells.

To test this hypothesis, a rescue experiment of phagocytosis was carried out by incubating the haemocytes obtained from silenced larvae (unable to perform phagocytosis) in the plasma from control larvae: this restored phagocytosis of bacteria *in vitro* (*t* = 11.67, *P*<0.0001, n = 14, df = 26) (Fig 10C). In contrast, haemocytes obtained from control larvae did not show phagocytic activity *in vitro* when resuspended in the plasma obtained from silenced larvae, which contained much lower levels of *Sl* gasmin. This experiment allows to conclude that (1) *Sl* gasmin is an essential element required for pathogen recognition and subsequent phagocytosis by haemocytes, and (2) the haemocytes of both control and silenced larvae are fully functional.

To further clarify the role of *Sl* gasmin in the haemolymph, the hypothesis of a direct interaction between the protein and the pathogen was investigated by a proteolytic shaving approach coupled with LC-MRM/MS, which has previously been successfully used to identify host proteins interacting with invading bacteria [46]. For this purpose, intact *E*. *coli* cells were incubated with the plasma of immune primed *S*. *littoralis* larvae, which contain high levels of *Sl* gasmin. After incubation, the bacterial surface was proteolytically shaved to release putative *Sl* gasmin peptides, demonstrating the occurrence of a direct interaction of the protein with bacterial cells. The released peptide mixtures were then analysed by LC-MRM/MS, monitoring specific peptides selected from *Sl* gasmin sequence (S2 Table). The level of *Sl* gasmin adhering to *E*. *coli* cells was substantially and significantly higher (*t* = 7.969, *P*<0.0001, n = 5, df = 7) than that recorded in control plasma used for incubating the bacteria (Fig 10D). Hence, *Sl* gasmin present in the haemolymph plasma sticks on the surface of invading bacteria and is essential to mediate bacteria recognition and subsequent phagocytosis reaction by haemocytes. Bacteria incubated with buffer allowed the exclusion of false positive results.

## Discussion

A homologue of the bracoviral gene *BV2-5* was recently found in the genome of *S*. *exigua* (named *gasmin*) [25]. Here we have found an additional homologue of *BV2-5* in the genome of *S*. *littoralis* (*Sl gasmin*), and observed that is highly expressed in larval stages exposed to an immune challenge. The results of the present study, based on complementary functional and molecular evidence, unequivocally demonstrate that this gene encodes an opsonizing factor triggering a fast phagocytic response. As already suggested [25], *gasmin* has been likely acquired by a basal ancestor of *Spodoptera* genus and maintained in distant species which separated about 24 MYA (*S*. *littoralis* and *S*. *exigua*) [47]. This suggests that there is a strong positive selection favoring the fixation of this gene, which, however, cannot be found in some related species (*S*. *frugiperda*), where the gene has been lost during evolution [25]. The important immune function of this gene, acquired through a mechanism of HGT mediated by a bracovirus, which acts as a unique gene vector [6, 25], reinforces the defense toolkit of *S*. *littoralis*. The integrative properties of BV have likely allowed the horizontal transfer of this viral gene. Indeed, parasitism on non-host larvae escaping death and accidental integration of BV sequences into the germ line allowed the stable integration into the moth genome and its transfer over generations [25]. This is the likely scenario in which several bracovirus genes have been acquired by the monarch butterfly (*Danaus plexippus*), the silkworm (*Bombyx mori*), the beet armyworm (*S*. *exigua*) and the fall armyworm (*S*. *frugiperda*), even though an adaptive advantage for these recipient insects has not been unequivocally demonstrated [25, 30]. To broaden the survey of *gasmin*-like genes present in insects and bracoviruses, we retrieved all candidate sequences available in the public databases, which showed high similarity to *Sl gasmin* (in total 17). These genes show great variability both in bracoviruses and insects, and the phylogenetic analyses fail to conclusively resolve any relationships (particularly for the *S*. *litura* homologue). The topology of the recovered phylogenetic tree might be the result of HGT and gene loss events, or other large modifications which led to a rapid evolution of the gene under positive selection, as expected for transferred genes response to new genetic and environmental contexts [10]. The *gasmin* genes are no exceptions, as most of the retrieved sequences have largely diverged. However, the striking similarity and conservation of the genomic and protein sequences present in *S*. *littoralis* and *S*. *exigua* strongly suggest a common origin. Further sampling of virus and insect gasmin homologues would be required to conclusively resolve this issue.

Collectively, the experimental evidence we present on *Sl gasmin* indicates that its transcription in the haemocytes is rapidly boosted by immune challenge, and the encoded protein is an opsonizing factor. Once in the haemolymph, *Sl* gasmin provides a better protection by mediating a fast phagocytic reaction by the haemocytes, which is abolished by RNAi-mediated gene silencing. However, this evidence raises the question about the role of the viral homologue, which is nearly identical and likely functionally equivalent. We may reasonably speculate that in the case of parasitized host larvae, where the BV genes are actively expressed, the viral homologue of *Sl gasmin* may limit the risk of accidental bacterial infection, which would be detrimental both for the host and the developing parasitoid progeny. Indeed, 24 h after oviposition by *C*. *congregata*, the viral gene *BV2-5* is highly expressed in the fat body and in the haemocytes of parasitized *Manduca sexta* larvae, where, concurrently, antimicrobial peptides are up-regulated, while genes involved in the phenol oxidase cascade and cellular immune responses are strongly down-regulated [48, 49]. Then, our results and the evidence available in the literature both point out that the immunosuppression induced by the wasp is selective, and provides protection to its juveniles by disabling only the responses effective against large intruders, leaving unaltered or even potentiating the antimicrobial barriers, in order to prevent dangerous secondary infections by pathogens. This could have been one of the functional constraints driving the evolutionary pathway of integration of this gene in the BV genome. Indeed, reinforcing the clearing capacity of the host haemolymph by enhancing the phagocytic activity appears to be an effective strategy when, concurrently, other barriers are very rapidly disrupted by maternal secretions injected at the oviposition by the parasitic wasp. This is an interesting hypothesis worth of further research efforts. The effective importance *in vivo* of this mechanism of haemolymph clearance is supported by the reduced capacity to withstand *Bt*-induced septicaemia we observed in *S*. *littoralis* larvae exposed to RNAi-mediated silencing of *Sl gasmin*. This implies that the acquired opsonizing factor is important in the modulation of phagocytosis efficiency *in vivo*.

Phagocytosis is one of the first, most rapid and effective barriers of protection against microorganisms. Indeed, haemocytes immediately start their phagocytic activity in response to intrusion of pathogens into the haemolymph, and each cell is able to engulf hundreds of bacteria [41]. Phagocytosis initiation can be either direct, via specific haemocyte-surface receptors, or indirect, via opsonins that label pathogens, and thus make them recognizable by haemocyte-surface phagocytic receptors [40, 41, 50]. Opsonization factors in insects are still poorly studied [51–53]. In Diptera, opsonization-dependent phagocytosis is mainly mediated by thioester-containing proteins (TEP), a group of proteins that includes the α2-macroglobulins and complement factors that mediate phagocytosis in vertebrates, which have been reported both for *Anopheles* and *Drosophila* species [50–52, 54, 55]. TEP are proteins that specifically promote the phagocytosis of different sets of pathogens [52]. In Lepidoptera, several opsonins secreted by different tissues have been described [53, 56–58] and, in all cases, opsonization factors showed the capacity to bind a broad range of microbes, such as both Gram-positive and Gram-negative bacteria, and yeasts. Here we present evidence that *Sl* gasmin is an additional opsonization factor present in Lepidoptera, which has been transferred through a BV associated with a parasitic wasp attacking a non-permissive ancestor of *Spodoptera* species. We speculate that this passage of *Sl gasmin* confers effective and fast protection to the wasp eggs and juveniles soon after parasitization. This is relevant from a functional point of view, as the insertion of the ovipositor into the host body can be a route of infection, besides any other risk of accidental infection. A future comparative analysis of opsonizing factors active in the immune response by *Spodoptera* species lacking gasmin-like factors will likely shed light on the adaptive mechanisms that have favoured the fixation of this gene during evolution. We can reasonably speculate that the addition of gasmin to the array of pre-existing opsonizing factors results in a faster reaction and a broader capacity to engulf different types of microorganisms. These are likely possibilities worth of further research efforts.

The contrasting functional evidence provided for *S*. *exigua gasmin*, generated, as already said above, by a biased experimental approach [25, 30], is problematic to interpret both from a mechanistic and evolutionary point of view. It is not easy to imagine that a protein triggering cytoskeleton disruption associated with reduced viral infection does not affect a number of other functions, with possible trades-off counteracting the fixation of its coding gene. This seems to be the case, as the proposed putative benefit of protection against viruses brings in reduced protection against other pathogens; indeed, haemocytes from an European strain of *S*. *exigua*, which do not produce functional gasmin, as it bears a truncated *gasmin* gene, fails to engulf bacteria by phagocytosis when infected with the recombinant baculovirus expressing *gasmin* [30]. This does not fit with what we have observed in the present study and it is difficult to imagine that we have so different functions for proteins sharing a very high level of sequence identity. While we performed a functional analysis by RNAi mediated gene silencing *in vivo*, for *S*. *exigua* gasmin a recombinant baculovirus was used for *in vitro* functional studies in an insect population that does not have genes encoding gasmin. This could have generated uncontrolled phenotypic responses, partly due to the concurrent effect of viral infection and the overexpression of a heterologous protein, that we do not even know if it is secreted outside of the infected cells [25, 30]. Moreover, data derived from these *in vitro* studies are somewhat difficult to interpret from an *in vivo* functional perspective. In particular, it is not easy to justify why *gasmin*, considered as an immune disrupter, is up-regulated in response to an immune challenge [30]; conversely, the results we present here are highly consistent with the proper immune role of *Sl gasmin*.

In conclusion, our results demonstrate that in insects an immune function has been reinforced by HGT of a viral gene. The route of acquisition of the immune gene *Sl gasmin*, which is mediated by a viral symbiont of a parasitoid wasp attacking moth larvae, sheds light on a completely novel evolutionary pathway [6, 25]. The integrative properties of BV, which are powerful natural genetic engineers, pave the way for the transfer of sequences among completely different evolutionary lineages. In the case of immune genes, these may undergo intense selection in the context of the complex immune interplay among parasitic wasps harbouring the BV, their hosts and associated microbiota, eventually favouring the most efficient traits conferring protection to the system. The “jump” of this genetic material in moth species generates the addition of a very effective function, paradoxically obtained with the help of a parasitoid. Then our study demonstrates that, in multicellular organisms, essential physiological functions can be acquired and/or shaped by HGT and do not always derive from evolution of pre-existing genes.

## Materials and methods

### Insects

*S*. *littoralis* larvae were reared on artificial diet (47.3 g/l wheat germ, 67.3 g/l brewer’s yeast, 189 g/l corn meal, 6.8 g/l ascorbic acid, 0.75 g/l cholesterol, 0.5 g/l propyl 4-hydroxybenzoate, 3 g/l methyl 4-hydroxybenzoate, 1.3 g/l wheat germ oil, 33.8 g/l agar and 3 g/l vitamin mix (1.2 g/Kg vitamin B1, 2.6 g/Kg vitamin B2, 2.5 g/Kg vitamin B6, 40 g/Kg choline, 10 g/Kg pantothenic acid, 32 g/Kg inositol, 0.25 g/Kg biotin, 2.5 g/Kg folic acid, 5 g/Kg 4-aminobenzoic acid, 0.5 mg/Kg vitamin B12, 10 g/Kg glutathione, 2.1 g/Kg vitamin A, 0.25 g/Kg vitamin D3, 24 g/Kg vitamin E, 0.25 g/Kg vitamin K, 25 g/Kg vitamin C in dextrose)), at 25 ± 1°C, 70 ± 5% R.H., and under a 16:8 h light/dark period.

### Tissue sample collection and RNA/DNA extraction

*S*. *littoralis* larvae were anaesthetized on ice and surface-sterilized with 70% ethanol prior to dissection. Larval haemolymph was collected from a cut of the leg and haemocytes were separated from plasma by centrifugation for 5 min, at 500 × g, at 4°C. Midgut and fat body were isolated after cutting the larval body lengthwise, and the remaining body carcass separately collected. These samples (i.e., haemocytes, midgut, fat body and carcass) were immediately transferred into TRIzol reagent (Life Technologies, Carlsbad, CA, USA) and kept at -80°C until total RNA extraction, that was performed according to manufacturer’s instructions. DNA was extracted from haemocytes using the protocol described elsewhere [59], with minor modifications. The concentration of extracted RNA or DNA was assessed by measuring the absorbance at 260 nm, with a Varioskan Flash Multimode Reader (Thermo Scientific, Waltham, MA, USA), and sample purity was evaluated by assessing 260/280 nm absorbance ratio. RNA quality was checked by electrophoresis on 1% agarose gel.

### *Sl gasmin* cloning and bioinformatics analysis

A partial *Sl gasmin* cDNA (FQ973054.1) was identified by BLAST analysis [60, 61] in a public database of expressed sequence tags (EST) from *S*. *littoralis* female antenna, using as query the full length cDNA sequence of *S*. *exigua gasmin* (KP406767.1).

To isolate *Sl gasmin* ORF, total RNA extracted from haemocytes of *S*. *littoralis* 6^th^ instar larvae was subjected to retro-transcription (Ambion RETROscript^®^ kit—Life Technologies). Given the very high level of sequence identity to *gasmin* (Fig 1), a cDNA was obtained by PCR, using Phusion High-Fidelity DNA Polymerase (Fisher Scientific, Pittsburgh, PA, USA) and primers designed to amplify the whole *gasmin* ORF (*gasmin* ORF forward primer ATGTTGCCTATTACCATACTAACG, *gasmin* ORF reverse primer ATACTGGAATTGGACATATTTGAGC). PCR conditions were programmed for 30 s at 98°C; 40 cycles of [10 s 98°C, 30 s 60°C, 1 min 72°C] and 15 min at 72°C. After amplification, the obtained PCR product was separated by gel electrophoresis and the visible band of the expected size was purified with a Quick gel extraction & PCR purification COMBO Kit (Invitrogen, Carlsbad, CA, USA). The PCR product was cloned into Zero-Blunt TOPO vector (Zero-Blunt TOPO PCR Cloning Kit, Invitrogen), according to the manufacturer’s instructions. After transformation of One Shot TOP10 chemically competent *E*. *coli* cells (Invitrogen), the transformants were incubated overnight at 37°C on LB plates containing 50 μg/ml kanamycin. Bacterial colonies containing the fragment of the appropriate size were selected by colony PCR, using Phusion High-Fidelity DNA Polymerase (Fisher Scientific) and M13 Forward (-20)/M13 reverse primers (Invitrogen), and grown overnight in LB medium containing 50 μg/ml kanamycin. The plasmid DNA was extracted from 4 ml of bacterial culture using a Charge Switch-Pro plasmid miniprep Kit (Invitrogen), as instructed by the manufacturer and sequenced.

The presence of an intron into *Sl gasmin* sequence was determined by PCR amplification of the total DNA extracted from *S*. *littoralis* haemocytes using Phusion High-Fidelity DNA Polymerase (Thermo Fischer Scientific) with specific primers (*gasmin* ORF forward primer ATGTTGCCTATTACCATACTAACG; *gasmin* intron reverse primer CAGGTGTCCGCATTCCACTGA). The length of PCR products was checked by electrophoresis on 1% agarose gels, before sequencing.

Extensive similarity searches of complete and high-throughput genome sequence databases hosted by NCBI using TBLASTN, as well as of non-redundant protein databases (BLASTP), using the inferred *S*. *exigua* and *S*. *littoralis* protein sequences, allowed the identification of numerous potential homologues. These were manually annotated to identify probable coding and intronic regions. Preliminary alignments of inferred amino acid sequences (351 aa) were performed using Muscle [62] and manually filtered to identify homologues, which could be aligned essentially contiguously with the *Spodoptera* protein sequences. Alignments were manually refined, and ambiguously aligned regions were excluded using the program GBlocks [63], leaving XX amino acid positions for phylogenetic reconstruction using PhyML [64] as implemented in the program SEAVIEW [65], under the JTT amino acid substitution matrix, with 4 gamma distributed substitution rate categories (see S2 File). Bootstrap proportions were estimated using parameters optimized on the original ML tree (rate distribution parameter (alpha) = 1.45).

To identify signatures of selection the FEL (Fixed Effects Likelihood) method was used [66], implemented at the DataMonkey website (http://datamonkey.org/) [67]. Nucleotide sequences of *S*. *exigua*, *S*. *littoralis* and *C*. *congregata* virus circle 25, including the intron, were aligned and the alignment was manually curated to maintain in-frame codon alignment. Selection was tested in the *S*. *exigua*—*S*. *littoralis* branches.

### *Sl gasmin* expression analysis by qRT-PCR

Total RNA used for transcriptional analysis was isolated as described above. Relative expression of studied genes was measured by one-step qRT-PCR, using the SYBR Green PCR Kit (Applied Biosystems, Carlsbad, CA, USA), according to the manufacturer’s instructions. *S*. *littoralis β-actin* gene (Z46873) was used as endogenous control for RNA loading. Primer Express 1.0 software (Applied Biosystems) was used to design the primers used (S3 Table). Relative gene expression data were analyzed using the ΔΔCt method [68–70]. qRT-PCR for measurement of *Sl gasmin* expression was carried out using specific primers (S3 Table), designed to detect a region of *Sl gasmin* mRNA not included in the sequence targeted by the dsRNA (see “dsRNA synthesis” paragraph). For validation of the ΔΔCt method, the difference between the Ct value of *Sl gasmin* and the Ct value of *β-actin* transcripts [ΔCt = Ct (*Sl gasmin*)-Ct (*β-actin*)] was plotted versus the log of ten-fold serial dilutions (2000, 200, 20, 2 and 0.2 ng) of the purified RNA samples. The plot of log total RNA input versus ΔCt displayed a slope less than 0.1 (Y = 1.149+0.0133X, R^2^ = 0.0493), indicating that the efficiencies of the two amplicons were approximately equal.

### Expression profiles of *Sl gasmin* in response to different pathogens

To analyze *Sl gasmin* expression in response to microbial challenge, *S*. *littoralis* 5^th^ instar larvae, surface-sterilized with 70% ethanol and chilled on ice, received an intra-haemocoelic injection of 2 × 10^7^ *E*. *coli*, 3 × 10^8^ *S*. *aureus* or 2 × 10^7^ *S*. *cerevisiae* cells, suspended in 5 μl of PBS (Phosphate Buffered Saline: 137 mM NaCl, 2.7 mM KCl, 10 mM phosphate buffer, pH 7.4). Injections were performed through the neck membrane, using a Hamilton Microliter 1701RN syringe (10 μl, gauge 26s, length 51 mm, needle 3). At the injection and at different time points after injection, experimental larvae (n = 12 for each experimental treatment) were dissected and haemocytes were collected and processed for total RNA extraction as described above; the relative expression of *Sl gasmin* was assessed by qRT-PCR.

### dsRNA synthesis

Total RNA extracted from haemocytes of *S*. *littoralis* 6^th^ instar larvae was retro-transcribed (Ambion RETROscript kit, Life Technologies) and a 789 bp long *Sl gasmin* cDNA fragment was obtained by PCR, using the *Sl gasmin* dsRNA forward primer (GCCGGCATGTTGTCTATTACC) in combination with the *Sl gasmin* dsRNA reverse primer (TCCTTCCAGCTTCTGAGTCA). This cDNA fragment was used as template for a nested-PCR reaction, performed with primers containing at their 5’ ends the T7 polymerase promoter sequence (T7-*Sl gasmin* forward TAATACGACTCACTATAGGGAG-TTCGAGGATACAAGCAGAG; T7-*Sl gasmin* reverse TAATACGACTCACTATAGGGAG-GGATGCTCAGGATATCTGTTAC). The resulting PCR product was used as template to synthesize *Sl gasmin* dsRNA (522 bp long), using the Ambion MEGAscript RNAi Kit (Life Technologies), according to the manufacturer’s instructions. Control dsRNA, 500 bp long, was obtained from a control template supplied by the kit used. dsRNA preparations were quantified by measuring their absorbance at 260 nm with a Varioskan Flash Multimode Reader, and purity was evaluated by assessing 260/280 nm absorbance ratios. Products were run on 1% agarose gels to confirm their integrity.

### Administration of dsRNA to *S*. *littoralis* larvae and silencing of *Sl gasmin*

*S*. *littoralis* 4^th^ instar larvae (1^st^ day) were anaesthetized on ice and 1 μl of *Sl gasmin* dsRNA or control dsRNA (see above), dissolved in PBS, was poured into the lumen of the foregut by means of a Hamilton Microliter 1701RN syringe (10 μl, gauge 26s, length 51 mm, needle 2). dsRNA treatments consisted of one oral administration of 150 ng per day, for 3 days (from 4^th^ to 5^th^ instar). After the last dsRNA administration and prior to any experiment, haemocytes from 3–4 treated larvae were used for qRT-PCR analysis, to confirm the occurrence of gene silencing.

### Cry1Ca bioassay and assessment of bacterial load

Purified Cry1Ca protein was produced in a recombinant *B*. *thuringiensis* strain EG1081 (Ecogen Inc.). Prior to use, Cry1Ca was dialyzed overnight, at 4°C in 50 mM sodium carbonate buffer, pH 9.0. After dialysis, toxin concentration was determined by the Bradford assay [71], using bovine serum albumin as standard.

Silenced and control larvae were singly isolated in multi-well plastic trays (Bio-Ba-32, Color-Dec, Italy), containing artificial diet, covered with perforated plastic lids (Bio-Cv-4, Color-Dec), and maintained under the rearing conditions reported above. For the first 3 days, the upper surface (1 cm^2^) of the artificial diet (0.3 cm^3^) was uniformly overlaid with 50 μl of purified Cry1Ca toxin, dissolved in 50 mM sodium carbonate buffer at pH 9.0. Control larvae were reared on artificial diet overlaid with 50 μl sodium carbonate buffer. Experimental larvae were maintained on artificial diet, replaced every 24 h, and daily inspected for survival, until pupation. To determine the 50% lethal concentration (LC_50_) of Cry1Ca toxin, the bioassay was carried out at 5 different concentrations of toxin and using 16 larvae for each experimental condition and control. Probit analysis [72], to determine LC_50_ values at day 10, 90% fiducial limits and toxicity increase ratio (TI) for each experimental treatment, was performed with the POLO-PC program (LeOra Software, Berkeley, CA).

The assessment of the bacterial loads was performed as previously described [32]. Briefly, 6 h after the last *Sl gasmin* or *GFP* dsRNA administration, newly molted 5_th_ instar larvae of *S*. *littoralis*, were exposed for 3 days to 2.7 μg/cm^2^ (corresponding to the LC_50_ of Cry1Ca in *Sl gasmin* silenced larvae). Both experimental groups included internal controls maintained on a toxin-free diet. On day 7, larvae were transferred to an untreated diet and, 24 h later, the midgut and haemolymph were separately collected under a horizontal laminar flow hood as described above. Experimental samples were obtained by pooling 10 larvae. The experiment was repeated 3 times. Changes over time in the relative bacterial load in the midgut (n = 7 for each sampling point) and haemolymph (n = 8 for each sampling point) samples were determined by qRT-PCR, measuring the transcript level of *16S rRNA* (AJ567606.1) to assess the impact of *Bt* toxin and *Sl gasmin* silencing on bacterial proliferation. The qRT-PCR was performed as described above, using *S*. *littoralis β-actin* as reporter gene (primers used are reported in S3 Table).

### Cellular and humoral immune assays

The impact of gene silencing on cellular immune responses was assessed by scoring its effect on encapsulation, nodulation and phagocytosis. Encapsulation and nodulation responses were assessed as previously described [31, 32]. CM Sepharose fast flow chromatography beads (Pharmacia), suspended in PBS, were injected into the haemocoel of *S*. *littoralis* larvae using a Hamilton Microliter 1702 RN syringe (25 μl, gauge 22s, length 55 mm, needle 3). After 24 h, beads were recovered upon larval dissection and scored to evaluate their encapsulation rate, which was expressed with the encapsulation index (E.I. = [Σ (encapsulation degree × total beads of this degree)/ total beads × 4] × 100), that takes into account both the encapsulation degree of each recovered bead (0—no cells adherent to the beads, 1—up to 10 adherent cells, 2—more than 10 adherent cells but no complete layer around the bead, 3—one or more complete layers without melanization, 4—one or more complete layers with melanization) and the relative abundance of beads with a given encapsulation degree [73]. For the nodulation assay, 12 h after the last dsRNA administration, *S*. *littoralis* larvae, surface-sterilized with 70% ethanol and chilled on ice, received an intra-haemocoelic injection of 5 μl of a PBS suspension of 2 × 10^6^ *E*. *coli* cells, or 2 × 10^7^ *S*. *cerevisiae* cells. Injections were performed through the neck membrane, using a Hamilton 1701 RN SYR (10 μl, 26s gauge, 55 mm long, point style 3). A thoracic leg was cut 18 h after injection, and the exuding haemolymph was collected and immediately diluted into an equal volume of ice-cold MEAD anticoagulant buffer (98 mM NaOH, 145 mM NaCl, 17 mM EDTA, and 41 mM citric acid, pH 4.5). The haemocyte nodules occurring in the haemolymph samples were counted under a light transmitted microscope at 400× magnification (Axioskop 20, Carl Zeiss Microscopy, Germany), using a Bürker chamber. When an intense immune response gave rise to large aggregates of nodules difficult to count separately, the number of distinct nodules observed was arbitrarily doubled, because the percentage of nonwhite pixels measured (ZEN software; Carl Zeiss Microscopy) on the large aggregates was on average twice that measured on a bright-microscopy field containing discrete nodules and free haemocytes.

To measure phagocytosis competence of *S*. *littoralis* haemocytes, an *in vitro* assay was performed as described in [32] with minor modifications. Briefly, haemolymph samples were collected from a cut of the leg into ice-cold PBS (1:1 v/v) and added with an equal volume of a PBS suspension of 2 × 10^6^ fluorescein conjugated *E*. *coli* cells (K-12 strain BioParticles, fluorescein conjugate, Invitrogen) or 2 × 10^7^ *S*. *aureus* (Wood strain, BioParticles fluorescein conjugate, Invitrogen). After incubation with *E*. *coli* (10 min) or with *S*. *aureus* (30 min), samples were loaded into a Bürker chamber, where total and fluorescent haemocytes were counted under a fluorescence microscope (Axioskop 20). Prior starting incubation experiments, vital staining with trypan blue was used to routinely check the viability of collected haemocytes. A haemolymph aliquot was mixed with 0.4% (w/v) trypan blue (2:1 v/v), prior to count viable and dead cells under a light transmitted microscope (Axioskop 20), using a Bürker chamber. Haemocyte samples with a viability rate lower than 98% were discarded.

For rescue experiments with haemocytes from silenced larvae, haemolymph samples were extracted from *S*. *littoralis* larvae chilled on ice, 24 h after the last dsRNA administration (*Sl gasmin* dsRNA or control dsRNA). Samples were centrifuged 5 min at 500 × g, at 4°C. The plasma was kept on ice, haemocytes were resuspended in PBS and centrifuged as previously described. PBS was then removed and haemocytes from larvae treated with *Sl gasmin* dsRNA were resuspended in the plasma isolated from larvae treated with control dsRNA, while haemocytes from larvae treated with control dsRNA were resuspended in the plasma isolated from larvae treated with *Sl gasmin* dsRNA. Then, the phagocytosis by haemocytes was evaluated as described above.

The humoral immune response, as affected by gene silencing, was assessed by measuring the transcript level of genes encoding antimicrobial peptides and lysozyme, in response to injections of different microorganisms, as previously described [32]. Briefly, 6 h after the last dsRNA administration, *S*. *littoralis* larvae, surface-sterilized with 70% ethanol and chilled on ice, received an intra-haemocoelic injection of 2 × 10^7^ *E*. *coli* or *S*. *aureus* cells, or 3 × 10^8^ *S*. *cerevisiae* cells, suspended in 5 μl of PBS. Injections were performed through the neck membrane with a Hamilton 1701 RN SYR (10 μl, gauge 26s, length 55 mm, needle 3). At the time of injection and 18 h after injection, larvae (n = 8 for each experimental sample) were dissected and haemocytes, midgut, and fat body were collected and processed for total RNA extraction, as described above. The relative expression of *attacin 1* (FQ971100.1), *gloverin* (FQ965511.1), and *lysozyme 1a* (FQ961692.1) were thus assessed by q-RT-PCR as described above. Primers used are reported in S3 Table.

### Detection of actin filaments in haemocytes

Newly moulted 5^th^ instar larvae of *S*. *littoralis*, treated with *Sl gasmin* dsRNA or control dsRNA, as described above, were surface-sterilized with 70% ethanol and chilled on ice. Larval haemolymph from individual larvae was collected from a cut of the leg and placed on glass slides for 10 min, to allow the haemocytes to settle and attach to the glass. Haemolymph was then carefully removed and haemocytes rinsed 3 times with PBS. Attached cells were fixed for 10 min in 4% paraformaldehyde in PBS, washed 3 times in PBS and permeabilized for 4 min with 0.1% Triton-X100 in PBS. Haemocytes were washed 3 times in PBS and then incubated for 20 min with 4 μg/ml TRITC-phalloidin (Tetramethylrhodamine B isothiocyanate-phalloidin). After 3 rinses in PBS, the samples were mounted in Vectashield Mounting Medium with DAPI (Vector Laboratories) and examined under a fluorescence microscope (ZEISS Axiophot 2 epifluorescence microscope). The observations have been performed in 3 different experiments and considering at least 10 randomly selected microscopic fields for each experimental condition.

### Multiple reaction monitoring (MRM) targeted proteomic approach

To detect the presence of *Sl* gasmin in the plasma, haemolymph was centrifuged as described above to remove the haemocytes, and the supernatant (plasma) was stored at -80°C. Samples were then dissolved in denaturant buffer (6 M urea, 10 mM EDTA, 300 mM Tris, pH 8.0) containing dithiothreitol (10-fold molar excess on the Cys residues) at 37 °C for 2 h, before the addition of iodoacetamide (IAM) to perform carboamidomethylation, using 5-fold molar excess of alkylating agent on thiol residues. The mixture was incubated in the dark at room temperature for 30 minutes and the product was purified by Chloroform/Methanol/H_2_O precipitation. Supernatants were removed and the pellets were dried. Digestion of the protein mixture was carried out in 10 mM ammonium bicarbonate (AMBIC), using trypsin at a 50:1 protein:enzyme mass ratio. The samples were incubated at 37°C for 16 h and dried after acidification (10% HCOOH in water). To eliminate any impurity, samples were suspended in 200 μl of 100 mM AMBIC, filtrated by centrifugal filter units (0.22 μm) and dried in a speed-vac concentrator. Samples were evaporated and suspended in 50 μl of 0.1% HCOOH in water. Peptide mixtures were analyzed by LC-MRM/MS analysis using a Xevo TQ-S (Waters, Milford, MA, USA) with an IonKey UPLC Microflow Source coupled to an UPLC Acquity System (Waters), using an IonKey device. For each run, 1 μl peptide mixture was separated on a TS3 1.0 mm × 150 mm analytical RP column (Waters) at 60°C, with a flow rate of 3 μl/min using 0.1% HCOOH in water (LC-MS grade) as eluent A, and 0.1% HCOOH in acetonitrile as eluent B. Peptides were eluted (starting 1 min after injection) with a linear gradient of eluent B in A, from 7% to 95% in 55 min. The column was re-equilibrated at initial conditions for 4 min. The MRM mass spectrometric analyses were performed in positive ion mode using a MRM detection window of 0.5–1.6 min per peptide; the duty cycle was set to automatic and dwell times were minimal 5 ms. Cone voltage was set to 35V. The selected transitions and the collision energy for each *Sl* gasmin peptide are reported in S2 Table.

To determine whether *Sl* gasmin is able to bind to the surface of bacteria, plasma samples obtained from *S*. *littoralis* 5^th^ instar larvae (n = 20) were added to an equal volume of MEAD and incubated with an equal volume of *E*. *coli* suspension in PBS (4 × 10^6^ cells for each μl of haemolymph) for 1 h. The suspension was then centrifuged for 10 min, at 12,000 × g, at 4°C and the pellet resuspended in 2 ml of 10 mM phosphate buffer, 45 mM NaCl, pH 7.4. Centrifugation and resuspension were repeated and the bacterial pellet as well as supernatants were frozen in liquid nitrogen and stored at -80°C until use. In control experiments bacteria were incubated with PBS and MEAD (1:1:1 v/v/v). Samples were submitted to reduction, alkylation and tryptic digestion as described above. After the preparation step they were processed and analyzed by LC-MRM/MS, as previously described. LC-MRM/MS analyses were performed on 3 technical replicates for each biological replicate and the average of these multiple measurements was used for data analysis. The data obtained represent the average value of total ion current associated to each transitions for the selected peptides.

### Reagents

Unless differently indicated, all reagents were provided by Sigma-Aldrich, Italy.

### Statistical analysis

Data were analyzed using Prism (GraphPad Software Inc. version 6.0b, San Diego, CA, USA) and SPSS (IBM SPSS Statistics, Version 21, Armonk, NY) software. The comparison between 2 experimental groups was done using the unpaired Student’s *t* test, while in the case of more than 2 experimental groups, One-Way ANOVA. Two-Way ANOVA was carried out on AMP and *lysozyme 1A* immune induction experiments, with RNAi treatment and bacterial injection as factors, while a Three-Way ANOVA was carried out for bacteria relative quantification, with dsRNA treatment, time and Cry1Ca toxin exposure as factors. When necessary transformation of data was carried out, to meet the assumption of normality. Levene’s test was carried out to test the homogeneity of variance. When significant effects were observed (*P* value<0.05), Bonferroni’s post-hoc test was used. When one of the assumptions was not met, even after the transformation of the data, Kruskal-Wallis one-way ANOVA (non-parametric ANOVA) test was employed.

## Supporting information

S1 Table Statistical analysis of the data reported in Figs 6 and 9. Statistical analysis performed on the relative quantification of bacterial load by qRT-PCR and the humoral immune response by *Spodoptera littoralis* larvae as affected by RNAi.
(DOCX)

S2 Table Mass spectral parameters for *Sl* gasmin peptides. Specific tryptic peptides from *Sl* gasmin sequence were selected for LC-MS/MS analyses in MRM mode. Individual transitions from the parent ions to the most intense fragments and the corresponding collision energies are reported.
(DOCX)

S3 Table Primers for qRT-PCR analyses. Sequences of the primers used for qRT-PCR analyses (F: Forward, R: Reverse).
(DOCX)

S1 File Protein sequences with high similarity to *Spodoptera littoralis* gasmin gene. Aligned potential homologue protein sequences retrieved form extensive similarity searches of complete and high-throughput genome sequence databases hosted by NCBI.
(TXT)

S2 File Log file of FEL (Fixed Effects Likelihood) analysis. Log of the FEL analysis aimed at identifying selective pressure on *Spodoptera littoralis* and *Spodoptera exigua* sequences. The program uses a maximum-likelihood approach to infer nonsynoymous (dN) and synonymous (dS) substitution rates on a per-site basis for a given coding alignment and corresponding phylogeny.
(TXT)
