# Supplementary material for: Evolution of an insect immune barrier through horizontal gene transfer mediated by a parasitic wasp
Source: PLoS Genet. 2019 Mar 5;15(3):e1007998. doi: 10.1371/journal.pgen.1007998 (PMC6420030; doi:10.1371/journal.pgen.1007998)
Supplement: S1 Table — Statistical analysis performed on the relative quantification of bacterial load by qRT-PCR and the humoral immune response by Spodoptera littoralis larvae as affected by RNAi. (DOCX) [file pgen.1007998.s001.docx]

**S1 Table. Statistical analysis of the data reported in figures 6 and 9.**

| **Figure 6** | | | |
| --- | --- | --- | --- |
| interaction | dsRNA treatment | Cry1Ca treatment | Time |
| Haemolymph (H) | | | |
| F_2,91_=126.99; *P*<0.0001 | F_1,91_=817.88; *P*<0.0001 | F_1,91_=4083.84; *P*<0.0001 | F_2,91_=785.37; *P*<0.0001 |
| Midgut (M) | | | |
| F_2,86_=0.157; *P*=0.855 | F_1,86_=0; *P*=0.998 | F_1,86_=0.898; *P*=0.346 | F_2,86_=1.528; *P*=0.223 |
| **Figure 9** | | | |
|  | interaction | dsRNA treatment | immune challenge |
| *attacin 1* | F_3,56_=0.007; *P*=0.999 | F_1,56_=0.178; *P*=0.675 | F_3,56_=33.80; *P*<0.0001 |
| *gloverin* | F_3,56_=0.505; *P*=0.681 | F_1,56_=0.011; *P*=0.916 | F_3,56_=146.90; *P*<0.0001 |
| *lysozyme 1a* | F_3,56_=0.218; *P*=0.884 | F_1,56_=1.697; *P*=0.198 | F_3,56_=23.60; *P*<0.0001 |
