# Supplementary material for: Evolution of an insect immune barrier through horizontal gene transfer mediated by a parasitic wasp
Source: PLoS Genet. 2019 Mar 5;15(3):e1007998. doi: 10.1371/journal.pgen.1007998 (PMC6420030; doi:10.1371/journal.pgen.1007998)
Supplement: S2 Table — Specific tryptic peptides from Sl gasmin sequence were selected for LC-MS/MS analyses in MRM mode. Individual transitions from the parent ions to the most intense fragments and the corresponding collision energies are reported. (DOCX) [file pgen.1007998.s002.docx]

**S2 Table. Mass spectral parameters for *Sl* gasmin peptides.** Specific tryptic peptides from *Sl* gasmin sequence were selected for LC-MS/MS analyses in MRM mode. Individual transitions from the parent ions to the most intense fragments and the corresponding collision energies are reported.

| **Peptide** | **Precursor Ion**  **(m/z)** | **Product Ions**  **(m/z)** | **Collision Energy (V)** |
| --- | --- | --- | --- |
| **R.TPAYK.N [35, 39]** | 290.16 | P [y4] - 478.27 A [y3] - 381.21 Y [y2] - 310.18 | 10 |
| **R.VIEALMR.Q [43, 49]** | 416.24 | I [y6] - 732.41 E [y5] - 619.32 A [y4] - 490.28 L [y3] - 419.24 | 14 |
| **K.LSSHDITSNR.T [96, 105]** | 565.28 | S [y9] - 1016.48  S [y8] - 929.44  H [y7] - 842.41  D [y6] - 705.35  I [y5] - 590.33 | 20 |
| **K.NDVYGSR.D [130, 136]** | 405.69 | D [y6] - 696.33 V [y5] - 581.30 Y [y4] - 482.24 | 14 |
| **K.YAQHGASITQIGNR.Y [171, 184]** | 758.39 | H [y11] - 1153.61 G [y10] - 1016.55  A [y9] - 959.53  S [y8] - 888.49  I [y7] - 801.46 | 27 |
| **R.EAYANK.H [306, 311]** | 348.17 | A [y5] - 566.29  Y [y4] - 495.26 | 12 |
| **K.YVQFQY. [339, 344]** | 424.20 | V [y5] - 684.34 Q [y4] - 585.27  F [y3] - 457.21 | 15 |
