# Supplementary material for: Evolution of an insect immune barrier through horizontal gene transfer mediated by a parasitic wasp
Source: PLoS Genet. 2019 Mar 5;15(3):e1007998. doi: 10.1371/journal.pgen.1007998 (PMC6420030; doi:10.1371/journal.pgen.1007998)
Supplement: S3 Table — Sequences of the primers used for qRT-PCR analyses (F: Forward, R: Reverse). (DOCX) [file pgen.1007998.s003.docx]

**S3 Table. Primers for qRT-PCR analyses (F: Forward, R: Reverse).**

| **Primer name** | **Sequence (5’-3’)** | **Accession numbers** |
| --- | --- | --- |
| *Sl gasmin* - F | AGTCGTTCAGAATGGTAACA | *Sl gasmin* FQ973054.1 |
| *Sl gasmin* - R | GACGCATTGAAGCCAATCAT |  |
| *β-actin* - F | CCGTCTTCCCATCCATCGT | *β-actin* Z46873 |
| *β-actin* - R | CCTTCTGACCCATACCAACCA |  |
| *Attacin 1* - F | CGTTCTTAGACCGCAAGGAC | *attacin 1* FQ971100.1 |
| *Attacin 1* - R | CACGGAAGTGGTCGGGCT |  |
| *Gloverin* - F | GGCAGCACGGACGATTCTT | *gloverin* FQ965511.1 |
| *Gloverin* - R | CCGAGGTTGGTGCTGTCTCCGTT |  |
| *Lysozyme* - F | ATGAGGGATTGGGTGTGCC | *lysozyme 1a* FQ961692.1 |
| *Lysozyme* - R | TGGAACAGGCCGTAGTCCCG |  |
| *16S Ribosomal RNA* - F | ACTCCTACGGGAGGCAGC | *E. coli* *16S rRNA* gene AJ567606.1 |
| *16S Ribosomal RNA* - R | ATTACCGCGGCTGCTGGC |  |
